# Data from a terminated study on iron oxide nanoparticle magnetic resonance imaging for head and neck tumors

**DOI:** 10.1038/s41597-020-0392-z

**Published:** 2020-02-21

**Authors:** Hesham Elhalawani, Musaddiq J. Awan, Yao Ding, Abdallah S. R. Mohamed, Ahmed K. Elsayes, Ibrahim Abu-Gheida, Jihong Wang, John Hazle, G. Brandon Gunn, Stephen Y. Lai, Steven J. Frank, Lawrence E. Ginsberg, David I. Rosenthal, Clifton D. Fuller

**Affiliations:** 1 Department of Radiation Oncology, The University of Texas MD Anderson Cancer Center, Houston, TX USA; 2 Department of Radiation Oncology, Medical College of Wisconsin, Wisconsin, USA; 3 MD Anderson Cancer Center UThealth Graduate School of Biomedical Sciences, Houston, TX USA; 4 Department of Radiation Physics, The University of Texas MD Anderson Cancer Center, Houston, TX USA; 5 Department of Imaging Physics, The University of Texas MD Anderson Cancer Center, Houston, TX USA; 6 Department of Diagnostic Radiology, The University of Texas MD Anderson Cancer Center, Houston, TX USA; 7 Department of Radiation Physics, Graduate School of Biomedical Sciences, MD Anderson Cancer Center, Houston, TX USA; 8 Department of Radiation Oncology, Burjeel Medical City, Abu-Dhabi, UAE

**Keywords:** Head and neck cancer, Diagnostic markers

## Abstract

Node positive head and neck squamous cell carcinomas (HNSCCs) patients exhibit worse outcomes in terms of regional neck control, risk for distant metastases and overall survival. Smaller non-palpable lymph nodes may be inflammatory or may harbor clinically occult metastases, a characterization that can be challenging to make using routine imaging modalities. Ferumoxytol has been previously investigated as an intra-tumoral contrast agent for magnetic resonance imaging (MRI) for intracranial malignancies and lymph node agent in prostate cancer. Hence, our group was motivated to carry out a prospective feasibility study to assess the feasibility of ferumoxytol dynamic contrast enhanced (DCE)-weighted MRI relative to that of gadolinium-based DCE-MRI for nodal and primary tumor imaging in patients with biopsy-proven node-positive HNSCC or melanoma. Although this institutional review board (IRB)-approved study was prematurely terminated because of an FDA black box warning, the investigators sought to curate and publish this unique dataset of matched clinical, and anatomical and DCE MRI data for the enrolled five patients to be available for scientists interested in molecular imaging.

## Abstract (summary)

**Table:** 

Measurement(s)	imaging assay • head and neck squamous cell carcinoma
Technology Type(s)	magnetic resonance imaging
Factor Type(s)	contrast agent
Sample Characteristic - Organism	Homo sapiens

Machine-accessible metadata file describing the reported data: 10.6084/m9.figshare.11409516

## Background & Summary

Neck nodal involvement in head and neck squamous cell carcinomas (HNSCCs) represents one of the most important features for head and neck malignancies risk stratification^1^. Pretreatment diagnosis of the neck disease now relies on computed tomography (CT) and positron-emission tomography fused with CT (PET/CT) that have many limitations leading to uncertainty and the potential for both over- and under- treatment of patients^2^. Given the current limitations of conventional imaging, enhanced imaging technologies are relevant to solve this unmet need. Recent data suggest that primary tumor assessment with dynamic contrast enhanced magnetic resonance imaging (DCE-MRI) may reveal quantitative assessments of the tumor environment that are germane to treatment selection and response evaluation^3^. Currently, gadolinium is the standard of care contrast agent for DCE-MRI^4^. However, a large array of potential MR contrast agents exists, including ultra-small superparamagnetic iron oxides (USPIO). The USPIO contrast-enhanced MR imaging has been previously shown to be promising as an avenue for differentiation of benign and malignant lymph nodes^5,6^.

Although a prospective trial in prostate cancer was successfully conducted using a dextran coated USPIO, also known as ferumoxtran-10 (Combidex, AMAG Pharmaceuticals, Inc. Lexington, MA), a Food and Drug Administration (FDA) Advisory Panel recommended against approval of this agent^7^. Afterwards, a derivative of ferumoxtran-10, ferumoxytol (Feraheme, AMAG Pharmaceuticals, Inc. Lexington, MA, US) was FDA-approved for iron replacement therapy in iron deficiency anemia in chronic kidney disease^8,9^. Ferumoxytol is a semi-synthetic, carbohydrate-coated, magnetic iron oxide preparation. This compound is taken up by normal lymph nodes and excluded from malignant nodal tissue^10^.

To date, no USPIO contrast agents were FDA-approved for the indication of head and neck imaging. More interestingly, no prospective data existed regarding ferumoxytol for evaluation of lymph node involvement in HNSCC compared to other imaging methodologies. Hence, our group was motivated to carry out a feasibility study of USPIO DCE-weighted MRI for primary and nodal tumor in locally advanced HNSCCs relative to that of gadolinium-based DCE-MRI. Additionally, it was our aim to investigate the optimum dose, timing, or sequence parameters of MR tumor imaging in HNSCC or melanoma with ferumoxytol.

However, during the course of this IRB-approved study, after 5 patients had enrolled and received MRIs as specified, with no demonstrable toxicities observed, a series of 79 cases of anaphylactic reactions associated with commercial ferumoxytol administration in its listed FDA indication for iron deficiency anemia were identified using the FDA adverse event reporting system database. Patients’ ages ranged from 19 to 96, and almost 50% of all cases reported the anaphylactic reactions occurring with the first dose of ferumoxytol. Of the 79 cases, 18 were fatal despite immediate medical intervention and emergency resuscitation attempts. Consequently, given the potential risk to patients in a Phase 0/1 feasibility trial designed to minimize risk, the investigators discontinued patient accrual and study conduction pursuant to the FDA black box warning against ferumoxytol issued on March 30, 2015^11^.

Notably, however, in the interval since closing the trial, a randomized safety assessment for the FDA approved indication showed less than 1% incidence of moderate‐to‐severe toxicities (including anaphylaxis, or moderate‐to‐severe hypotension) within 5 weeks of ferumoxytol administration^12^; an update of 217 consecutive patients with wide range of ages, comorbid conditions and kidney functions given ferumoxytol specifically for MRI applications with no observed adverse events^13^; and a report of 69 patients receiving 85 ferumoxytol injections for MRI with 4 reported mild adverse events (2 episodes mild hypotension and one episode of nausea, and one episode warmness/erythema at the in injection site)^14^.

Consequently, as more recent enthusiasm for ferumoxytol as a potential (cancer) imaging agent has resurfaced, the potential utility of our extant, if limited, series prompts now public dissemination of the extant data from this terminated trial, and we have opted to curate and publish this unique dataset of matched clinical and imaging data for these 5 patients^15^. To our knowledge, this is the first and only extant matched USPIO contrast-enhanced MRI scans –among other structural sequences- and clinical data of HNSCC patients where scans were acquired serially prior to radiotherapy (RT) course. This dataset represents a unique repository resource for scientists interested in molecular imaging of underlying tumor biology for other USPIO molecules, as well as those who may find capacity for additional development in HNSCC for ferumoxytol for similar applications.

## Methods

### Study conception and initiation

Then-current leadership of the licensing vendor for Feraheme has explicitly stated during study development in 2011–2012, that the vendor has no intent or desire to investigate imaging applications *in vivo*. For this reason, Ed Neuwelt, MD at Oregon Health and Science University acquired orphan drug designation for Ferumoxytol for use in MRI imaging of brain cancers (https://www.accessdata.fda.gov/scripts/opdlisting/oopd/detailedIndex.cfm?cfgridkey=338411). At the time of protocol approval, ferumoxytol had an excellent safety profile, and based on these rationales, MD Anderson Cancer Center approved regulatory exemption from the Investigational New Drug regulations, 21 CFR 312; as:

(1) The investigational drug is lawfully marketed in the United States; (2) The investigation [was] not intended to be reported to the FDA as a well-controlled study in support of a new indication for use of the drug product, but rather as a feasibility study; (3) The investigation [was] not intended to support a significant change in advertising to an existing lawfully marketed prescription drug product; (4) The investigation does not involve a route of administration or dosage level or use in a patient population or other factor that significantly increases the risks (or decreases the acceptability of the risks) associated with the use of the drug product; (5) The investigation [was] conducted in compliance with the requirements for institutional review set forth in FDA regulations 21 CFR 56, and requirements for informed consent as set forth in FDA regulations 21 CFR 50; 6) The investigation [was] conducted in compliance with FDA regulations 21 CFR 312.7: Promotion and charging for investigational drugs.

After approval, the protocol was initiated, activated for enrollment, posted on ClinicalTrials.Gov [ClinicalTrials.gov Identifier NCT01895829 (https://clinicaltrials.gov/ct2/show/NCT01895829)]^16^, and accrual commenced.

### Study population and eligibility criteria

This single institute feasibility study was planned to include untreated biopsy-proven HNSCC or melanoma with multiple malignant lymph nodes by imaging (clinical N2b or greater per the American Joint Committee on Caner (AJCC) and Union for International Cancer Control (UICC) cancer staging system, 7^th^ edition^17^ presenting at MD Anderson Cancer Center This study was approved by the MD Anderson clinical ethics committee. Written informed consents were signed by all participating patients. This trial was also registered in MD Anderson Cancer Center Clinical Trials database as Study #2012–1127.

Inclusion criteria included patients 18 years of age or older with histologically or cytologically confirmed HNSCC or melanoma; along with measurable clinical and/or radiographic poly-nodal disease defined as stage N2b, N2c or N3 disease with multiple involved lymph nodes per the AJCC cancer staging criteria. All patients received or were dispositioned to receive a PET/CT scan within two weeks of starting definitive therapy for their head and neck malignancy and their participation in this study.

Patients were excluded from the study if they have already undergone definitive resection, chemotherapy, or radiation treatment of their primary or nodal disease. Patients were also excluded if they did not give written, informed consent, were unable to undergo MR imaging, incapable of tolerating DCE-MRI, or having an estimated glomerular filtration rate (GFR) < 60 ml/min/1.73 m^2^. A key exclusion criterion was contraindications to iron supplementation, as hemochromatosis, colitis, history of GI bleeds, alcoholism, or liver disease. Patients that had iron overload and/or hypersensitivity to Feraheme were also excluded. Women of childbearing potential were also ruled out. Male patients were obligated to practice effective contraception throughout the study. Other exclusion criteria were the presence of any evidence of iron overload on pre-imaging laboratory studies, or any contraindications to gadolinium-based contrast agents or claustrophobia.

All patients were clinically evaluated and staged in the standard fashion via physical examination and routine radiographic studies including all of the following: contrast-enhanced CT of the head and neck, PET/CT of the head and neck and chest x-ray. Patients also underwent routine baseline assessment by Dental Oncology, Speech Pathology and Dietary Services. Inclusion and exclusion criteria are summarized as follows:

**Inclusion criteria:**

- Patients 18 years of age or older
- Histologically or cytologically confirmed head and neck squamous cell carcinoma or melanoma
- Measurable clinical and/or radiographic poly-nodal disease defined as stage N2b, N2c or N3 disease with multiple involved lymph nodes as defined by the AJCC cancer staging criteria
- Patients who have received or are dispositioned to receive a PET/CT scan within two weeks of starting definitive therapy for their head and neck malignancy and their participation in this study. This implies patients must receive a PET/CT to be eligible for the study.

**Exclusion criteria**

- Patients who have undergone definitive resection of their primary or nodal disease as well as any chemotherapy or radiation therapy for their head and neck primary tumor.
- Patients unable or unwilling to give written, informed consent or to undergo MRI imaging.
- Women of childbearing potential (A woman of child-bearing potential is a sexually mature woman who has not undergone a hysterectomy or who has not been naturally postmenopausal for at least 24 consecutive months [i.e., who has had menses at any time in the preceding 24 consecutive months]). Male partners must practice effective contraception (oral, injectable, or implantable hormonal contraceptive; tubal ligation; intra-uterine device; barrier contraceptive with spermicide; or vasectomized partner) throughout the study.
- Patients unable to tolerate DCE-MRI or having an estimated GFR < 60 ml/min/1.73 m^2^.
- Contraindications to iron supplementation include hemochromatosis, colitis, and history of GI bleeds, alcoholism, or liver disease. Patients that have iron overload and/or hypersensitivity to Feraheme (brand name for Ferumoxytol), may consequently react negatively to it or anything within it. Consequently, we patients who had symptoms or signs that might be caused by iron overload were excluded. These include patients with (unexplained): arthritis (including premature osteoarthritis), congestive heart failure or cardiomyopathy, adult-onset diabetes, secondary hypogonadism, increased skin pigmentation, or patients with persistently elevated serum ferritin not explained by an underlying inflammatory/systemic disease, unless these patients demonstrate a fasting transferring saturation ≤ 0.45.
- Patients with any evidence of iron overload on pre-imaging laboratory studies.
- Patients with any contraindications to gadolinium-based contrast agents.
- Patients with Claustrophobia.

### Patient demographics and clinical end points

Five male patients with oropharyngeal cancer (OPC) and a median age of 67 years (range: 52–78) were included. Patients’ demographic and clinical data are listed in Table 1, whereas Online-only Table 1 contains definitions of the provided clinical data attributes.

**Table 1:** Patients Demographics.

Patient ID	Sex	Age at diagnosis (years)	Race/Ethnicity	HPV status	Smoking status at diagnosis	Primary tumor subsite	TNM status (7^th^ edition)	AJCC (7^th^ edition)	Scan intervals (hours)
USPIO-001	Male	65	White	Positive	Former	Tonsil	T4N2bM0	IV	0-7-17
USPIO-002	Male	69	White	Positive	Never	Base of tongue	T3N2cM0	IV	0-6-17
USPIO-003	Male	67	White	Negative	Former	Tonsil	T1N2bM0	III	0-24-48
USPIO-004	Male	78	White	Unknown	Former	Tonsil	T1N2bM0	III	0-24-72
USPIO-005	Male	52	White	Positive	Current	Tonsil	T1N2bM0	III	0-24-72

AJCC: The American Joint Committee on Cancer; HPV: Human papillomavirus.

The patients’ demographic data encompass age, race or ethnicity, and sex. Disease characteristics include: the OPC subsite, and the TNM (tumor, node, metastasis) status, where the **T category** describes the size and extent of the initial (primary) tumor, per the AJCC/UICC cancer staging system, 7th edition (25) (https://cancerstaging.org/references-tools/Pages/What-is-Cancer-Staging.aspx). Correspondingly, the **N category** describes the extent to where the cancer reached the nearby lymph nodes, per the AJCC and UICC cancer staging system, 7th edition, along with the corresponding AJCC stage.

#### Study design

This study was anticipated as a Phase 0 Feasibility study of USPIO ferumoxytol as a DCE contrast agent in primary and nodal tumor perfusion imaging for HNSCC or melanoma. Twenty patients were originally planned to participate in this study, but only five patients were able to participate and got all scans done before the issuance of the FDA black box warning, and hence the premature closure of the trial. These patients were intended to be accrued to obtain two dynamic susceptibility contrast (DSC) or DCE perfusion MR scans, prior to any chemo or radiotherapy for their primary and nodal disease. At the time of the first scan, patients received the standard non-contrast MRI and a gadolinium-enhanced DCE-weighted MRI per standard institution protocols in immobilization position^18^. Immobilization entails that patients are unable to move; lying on their back with a bite block, a complete thermoplastic mask, and a posterior tailored head, neck, and shoulder mold for radiotherapy.

Afterwards, following an IV bolus Ferumoxytol injection, T1 and T2-weighted as well as multiparametric MRI scans in immobilization were acquired as Ferumoxytol acts as both a T1 and T2 agent in the tumor^19^. Later on, patients received a second and a third post-Ferumoxytol scans after various waiting intervals (hours to days) to assess the nodal conspicuity. Exact waiting times relative to initial Ferumoxytol injection are depicted in Table 1.

Safety monitoring included a 45-minute post-Ferumoxytol infusion observation with vital sign monitoring to assess for acute reactions. Serum iron was performed at baseline MRI, 48 hours post-infusion, and 1-month post-infusion. Patients returned for follow-up visits for these toxicity assessments as indicated by the same timeline.

### Imaging characteristics and MRI protocol

**Fig. 1:**
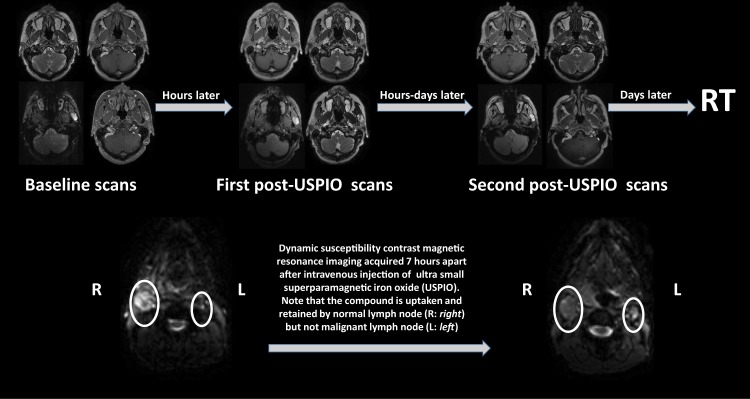
The scanning timeline and an illustration of pattern of enhancement of iron oxide nanoparticle magnetic resonance imaging.

Each patient received 3 anatomical and multi-parametric MRI scans the week before initiating RT. Figure 1 depicts the scanning timeline along with an illustration of how USPIO pattern of enhancement can discriminate benign and malignant lymph nodes. The interval between each scan and the following one ranged between 6 and 96 hours (Table 1). The scans were obtained using a 3.0 T Discovery 750 MRI scanner (GE Healthcare) with a flat insert table (GE Healthcare) and six-element flex coils. The exact same immobilization devices (individualized shoulder and neck mold, tailored head support, and bite block) were utilized in longitudinal scans to enhance co-registration of images and diminish physical motion (e.g., swallowing). This design structure was explained by Hollows et al. where it was shown that this setup with the bite block is much more reproducible than the usual setup^20^. Although this setup has not been proven to be reproducible yet, this enhanced reproducibility demonstrated at one institution would probably extend to other institutions^21^.

Thirty slices, 4 mm thick each, with a field of view of 25.6 cm, were chosen to screen the spatial region including the palatine process region cranially to the cricoid cartilage caudally for all scans. Before the DCE-weighted MRI scan, T1 mapping was done by utilizing six variable-flip-angle 3D spoiled gradient recalled echo sequences (SPRG) (flip angles: 2°, 5°, 10°, 15°, 20°, and 25°; repetition time/echo time, 6.1/2.1 ms; effective number of excitations, 0.7 (GE terminology, number of averages = percent sampling * number of signal averages/number of excitations); acquisition resolution, 2 mm × 2 mm × 4 mm; zero filling interpolation, × 2; scan time, 3 min). The DCE-MRI acquisition included a three-dimensional quick spoiled gradient recalled echo sequence to gain enough temporal resolution, contrast, and signal-to-noise ratio (SNR). These scan parameters were employed: a 15° flip angle; a repetition/echo time of 3.6/1.0 ms; 0.7 excitations; an acquisition resolution of 2 mm × 2 mm × 4 mm; a × 2 zero filling interpolation; 5:08 min scanning time; 5.5 s temporal resolution; 50 kHz bandwidth; an acceleration factor of 2; and 56 temporal. The DSC-MRI acquisition included a spin echo EPI sequence to gain enough temporal resolution (1.5 sec), contrast, and signal-to-noise ratio (SNR). These scan parameters were employed: a 45° flip angle; a repetition/echo time of 1500/14.2 ms; an acquisition resolution of 2 mm × 2 mm × 4 mm; 2:15 min scanning time; 1.5 s temporal resolution; 250 kHz bandwidth; an acceleration factor of 2; and 90 temporal.

The DCE/DSC MRI scan was initiated before, during, and following a bolus injection of 0.1 mmol/kg gadopentetate dimeglumine (Magnevist, Bayer Healthcare Pharmaceuticals) delivered at 3 ml/s followed by a 20 ml saline flush also delivered at 3 ml/s. An hour later, subjects received a dose of Ferumoxytol containing 512 mg elemental iron (the FDA package dose for iron deficiency anemia), injected as an IV bolus push as per package insert instructions. A Medrad Solaris MR-compatible power injector was used for all contrast agent injections in order to standardize the contrast agent administration. Scanner and acquisition parameters for various MRI sequences obtained are listed in Online-only Table 2.

### Data anonymization

We used the Clinical Trial Processor (CTP), developed by the Radiological Society of North America (RSNA) to anonymize the imaging DICOM files^22^. Data anonymization was completed in conformation with the HIPAA, as attributed by the DICOM standards committee Attribute Confidentiality Profile (DICOM PS 3.15: Appendix E), which explains the standardized methods and documentation for eradication of secured health records from DICOM images^23^. A final DICOM anonymization quality assurance was employed utilizing a software, ImageJ (https://imagej.nih.gov/ij/), which compiles all DICOM header tags for an image in a report that we later manually reviewed to ensure that ideal anonymization was achieved. The DICOM files were uploaded to VelocityAI™ 3.0.1 software, where the images were visually examined, to verify that our process of anonymization has not changed the spatial information encompassed in the DICOM header.

## Data Records

This head and neck cancer imaging dataset of 5 patients includes anonymized anatomical and multiparametric MRI sequences that are listed in Online-only Table 2. These scans were acquired at three different time points per patient; all prior to the initiation of radiotherapy course. Pertinent clinical meta-data files were also presented as.csv sheets, i.e., files with values separated by commas. This allows data to be put in a structured table format. CSVs are like spreadsheets with a .**csv** extension. The data records and supplemental descriptions of the clinical meta-data files are cited under figshare (10.6084/m9.figshare.c.4420499.v2)^24^. All images followed the standardized DICOM format and the images are labeled by anonymized patient ID numbers. Also, using the identifier, the Patient IDs can be cross-referenced to the data table ‘PatientID’ column.

**Fig. 2:**
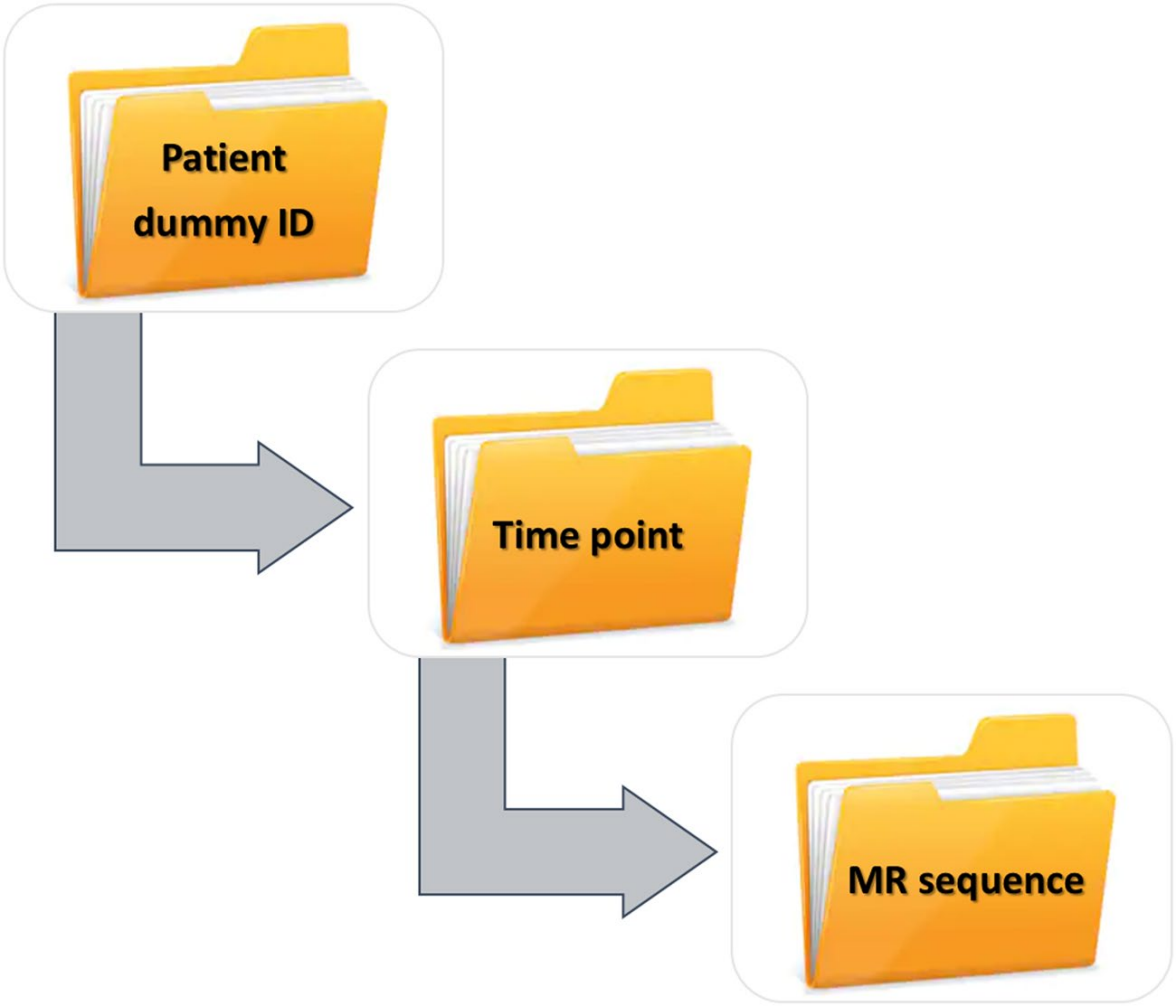
The directory structure of uploaded imaging dataset at figshare.

The imaging dataset is organized into 5 main folders. Each folder name represents individual patient’s ID that starts with ‘USPIO_’ followed by a number; 001 through 005. Under each folder, study participants can locate 3 folders, for all respective time points: baseline, first post-USPIO, and second post-USPIO scans. The next level of subfolders represent various MR sequences acquired at the corresponding time point (Fig. 2). The DICOM tag (0018,1314) classifies the flip angle that was used for the image slice in the variable flip angle images.

## Technical Validation

**ClinicStation (Electronic Medical Record System)**, a custom-built electronic medical record system by MDACC, that started in 1999 with subsequent significant improvement in 2007 that allowed further new capabilities, such as integrating research data and accessing data from virtually every electronic source within the institution, thus serving a great role in patient care and research. http://www.clinfowiki.org/wiki/index.php/ClinicStation.

## Usage Notes

We invite all interested researchers to download this dataset of matched clinical-imaging dataset including DCE/DSC- weighted perfusion MRI acquisitions in immobilization position using an under-investigated contrast agent in the setting of HNSCC imaging. Moreover, this data descriptor serves as a report for the only trial that prospectively investigated the feasibility of USPIO as a contrast agent for DCE-MRI acquisition purposes in the setting of head and neck cancer imaging.

## Data Availability

The Clinical Trial Processor (CTP) that was created by the Radiological Society of North America (RSNA) is available at: https://www.rsna.org/ctp.aspx. The **RSNA CTP** aligns meticulously to regulations for image anonymization, per the HIPAA Privacy Rule and the DICOM Working Group 18 Supplement 142. Being compatible with all commercially available picture archiving and communication systems (PACS), the RSNA CTP is designed to transport images to online data repositories where the anonymization process takes place. This program replaces patient tags in the DICOM files in a folder (and sub-folders) with anonymized tags that were assigned. **ImageJ**, a free software offered by the National Institutes of Health, USA, as a public domain Java processing program. The code for the software is accessible at: https://imagej.nih.gov/ij/.

## Online-only Tables

**Online-only Table 1:** Supplemental information about the clinical data.

Data category	Description
Patient ID	Numbers given randomly to the patient after anonymizing the DICOM PHI tag (0010,0020; Patient ID)
Sex	Patient’s sex
Age at diagnosis (years)	Patient’s age in years at the time of diagnosis
Race/Ethnicity	American Indian/Alaska Native, Asian, Black, Hispanic, White or NA (Not applicable)
HPV status	Human papilloma virus status
Smoking status at diagnosis	Never, current, or former
Primary tumor subsite	The subsite where the tumor originated within the head and neck, e.g., base of tongue (BOT) or tonsil
TNM status (7th edition)	The T category describes the original (primary) tumor, as regard its size and extent, per the 7th edition of American Joint Committee on Caner (AJCC) and Union for International Cancer Control (UICC) cancer staging system. It can be Tx, T1, T2, T3, or T4. The N category describes whether or not the cancer has reached nearby lymph nodes, per the AJCC and UICC cancer staging system. It can be N0, N1, N2a, N2b, N2c or N3. The M category tells whether there are distant metastases (spread of cancer to other parts of the body). It can be M0 or M1. https://cancerstaging.org/references-tools/Pages/What-is-Cancer-Staging.aspx
AJCC Stage	AJCC cancer stage. It can be 0, I, II, III or IV. https://cancerstaging.org/references-tools/Pages/What-is-Cancer-Staging.aspx.
Scan intervals (hours)	The interval between each scan and the following one. It ranged between 6 and 96 hours.

**Online-only Table 2:** Scanner and acquisition parameters for various MRI sequences.

Parameter	T1-baseline	T1 map	T1 + contrast	T2* GRE Baseline	T2* map	T2	T2 map	DCE (base line)	DSC (baseline)
Model	Discover 750	Discover 750	Discover 750	Discover 750	Discover 750	Discover 750	Discover 750	Discover 750	Discover 750
Slice Orientation	Axial	Axial	Axial	Axial	Axial	Axial	Axial	Axial	Axial
FOV	256 mm	256 mm	256 mm	256 mm	256 mm	256 mm	256 mm	256 mm	256 mm
Acquisition (Acq) Voxel Size	1 × 1 × 4	2 × 2 × 4	1 × 1 × 4	1 × 1 × 4	2 × 2 × 4	1 × 1 × 4	2 × 2 × 4	2 × 2 × 4	2 × 2 × 4
Acq Matrix	256 × 256	128 × 128	256 × 256	256 × 256	128 × 128	256 × 256	128 × 128	128 × 128	128 × 128
Reconstruction (Recon) Voxel Size	0.5 × 0.5 × 4	1 × 1 × 4	0.5 × 0.5 × 4	1 × 1 × 4	1 × 1 × 4	0.5 × 0.5 × 4	1 × 1 × 4	1 × 1 × 4	1 × 1 × 4
Recon Matrix	512 × 512	256 × 256	512 × 512	256 × 256	256 × 256	512 × 512	256 × 256	256 × 256	256 × 256
Parallel imaging	No	No	No	No	No	No	No	Yes, factor = 2	Yes, factor = 2
Fold-over Direction	AP	AP	AP	AP	AP	AP	AP	AP	AP
Qty Slices	30	No	30	30	30	30	30	30	30
Slice thickness	2.5 mm	4 mm	2.5 mm	3.5 mm	3.5 mm	2.5 mm	3.5 mm	4 mm	4 mm
Slice gap	1.5 mm	0	1.5 mm	0.5 mm	0.5 mm	1.5 mm	0.5 mm	0	0
Technique	SE	GRE/ SPRG	SE	GRE	GRE	SE	SE	GRE/ SPRG	SE
Fast Imaging Mode	FSE	None	FSE	None	None	FSE	None	None	EPI
Echoes	1	1	1	1	8	1	8	1	1
Flip Angle	90°	2°, 5°, 10°, 15°, 20°, 25°	90°	20°	20°	90°	90°	15°	45
TR	~631 ms	6.1 ms	~700 ms	500 ms	25 ms	3762 ms	850 ms	3.6 ms	1500 ms
Echo time	7.4 ms	2.1 ms	10 ms	10 ms	2.532,4.364,6.196,8.028,9.86,11.692,13.524,15.356 ms	97 ms	6.808,13.616,20.424,27.232,34.04,40.848,47.656,54.464 ms	1.0 ms	14.2 ms
Fat suppression	None	None	Yes, Fat Classic	None	None	None	None	None	Yes, Fat Classic
b-values	None	None	None	None	None	None	None	None	None
NEX	2	1	2	1	1	2	1	1	1
EPI factor/Echo train length	2		2	1	1	16	1	1	11
Bandwidth in frequency-direction	62.5 kHz	50 kHz	25 kHz	31.25 kHz	125 kHz	62.5 kHz	83 kHz	50 kHz	250 kHz
Scan Duration	2:10	6*30 sec	4:51	2:12	1:43	1:38	3:41	(56 dyn) 5:08	(90 dyn) 2:15

T2* GRE: gradient recalled echo; DSC: dynamic susceptibility contrast; DCE: dynamic contrast enhanced; FOV: field of view; AP: anteroposterior; SE: spin echo; FSE: fast spin echo; EPI: echo planar imaging; TR: time of repetition; NEX: number of excitations.
